# Possible association between ABCC8 C49620T polymorphism and type 2 diabetes in a Nigerian population

**DOI:** 10.1186/s12881-018-0601-1

**Published:** 2018-05-12

**Authors:** Godwill Azeh Engwa, Friday Nweke Nwalo, Claribel Chidimma Chikezie, Christie Oby Onyia, Opeolu Oyejide Ojo, Wilfred Fon Mbacham, Benjamin Ewa Ubi

**Affiliations:** 1 Biochemistry, Department of Chemical Sciences, Godfrey Okoye University, P.M.B 01014, Thinkers Corner, Enugu, Nigeria; 2 Department of Biotechnology, Federal University, Ndufu-Alike Ikwo (FUNAI), P.M.B. 1010, Abakaliki, Nigeria; 3 Department of Biotechnology, Godfrey Okoye University, P.M.B 01014, Thinkers Corner, Enugu, Nigeria; 4 Department of Biology, Chemistry and Forensic Science, School of Sciences, University of Wolverhampton, Wolverhampton, WV1 1LY UK; 5 Bioscience Research Education and Advisory Centre, Ibadan, Nigeria; 6 Laboratory for Public Health Research Biotechnologies, The Biotechnology Centre, University of Yaounde I, BP 8094 Yaounde, Cameroon; 7 Department of Biotechnology, Ebonyi State University, P.M.B. 53, Abakaliki, Nigeria

**Keywords:** Type 2 diabetes, ABCC8 gene, Genotyping, C49620T variant, Nigeria

## Abstract

**Background:**

The association between *ABCC8* gene C49620T polymorphism and type 2 diabetes (T2D) in populations of diverse ethnic backgrounds has been reported. However, such occurrence in an African population is yet to be established. This case-control study involving 73 T2D and 75 non-diabetic (ND) patients investigated the occurrence of this polymorphism among T2D patients in Nigeria and assessed its relationship with body lipids of patients.

**Methods:**

Demographic and clinical characteristics of patients were collected and lipid profile indices including total cholesterol (TC), triglyceride (TG), low density lipoprotein (LDL) and high density lipoprotein (HDL) were assayed. Restriction fragment length polymorphism-PCR (RFLP-PCR) was employed to genotype the *ABCC8-*C49620T polymorphism using *PstI* restriction enzyme.

**Results:**

This study revealed significantly (*p* < 0.05) higher prevalence of the T allele of the *ABCC8* gene in T2D patients (33.1%) compared to ND patients (28.0%). The mutant TT genotype was also higher than the CC and CT genotypes in T2D patients compared to ND patients but did not show any significant risk (*p*>0.05) of T2D for the unadjusted codominant, dominant and recessive models. Following age adjustment, the mutant genotypes (CT and TT) showed significant (*p*<0.05) risk of T2D for all the models with the recessive model presenting the greatest risk of T2D (OR: 2.39, 95% CI: 1.16-4.91, *p*<0.018). The TT genotype significantly (*p*<0.05) associated with high level of HDL and reduced levels of TC, TG and LDL in non-diabetic patients but was not associated with any of the demographic and clinical characteristics among T2D patients.

**Conclusions:**

ABCC8 C49620T polymorphism showed possible association with T2D marked by predominance of the mutant TT genotype in T2D patients. However, the relationship between TT genotype and lipid abnormalities for possible beneficial effect on people suffering from T2D is unclear.

## Background

Diabetes, one of the leading non-communicable diseases (NCD), has been recognized as an important cause of premature death and disability globally [1]. According to WHO, the global prevalence of the disease has risen from 108 million in the 1980s to a current figure of 422 million [2]. This increasing prevalence is becoming more prominent in middle or low income countries and T2D accounts for about 90% of all cases of diabetes [1].

T2D is a complex metabolic disease involving defective carbohydrates and lipids metabolism. This disease is characterized by high level of blood glucose due to impaired secretion of insulin and/or insulin resistance [3]. The principal role of insulin is to promote glucose uptake from blood or intercellular spaces into skeletal muscle cells and adipocytes or for transportation to other cells where glucose is needed [4]. Normally, pancreatic β-cells constantly synthesize insulin irrespective of the levels of blood glucose. Insulin, stored within vacuoles of β-cells is only secreted in response to elevated blood glucose levels. The secretion of insulin is controlled by the ATP-sensitive potassium (K_ATP_) channel. This channel consists of four pore forming subunits of Kir6.2 along with four sulfonylurea receptor (SUR) subunits [5]. SUR1 is the site where sulfonylurea proteins bind to trigger insulin secretion and it is coded by the ATP binding cassette, subfamily C, member 8 (ABCC8) gene.

The ABCC8 gene was identified as the locus for familial persistent hyperinsulinaemic hypoglycaemia of infancy, an autosomal recessive disorder characterized by unregulated insulin secretion [6]. Polymorphisms in the *ABCC8* gene has been associated with insulin response in Mexican American subjects [7] and T2D in French Canadians [8], but not in a Scandinavian cohort [9]. The C49620T variant of the ABCC8 gene is the most common polymorphism found to be associated with T2D [10]. Though association between C/T polymorphism of *ABCC8* gene on exon16–3 (rs1799854) has been shown to be associated with T2D in many populations groups [11], this association in people from African ethnic background living with T2D has not been established. Therefore, this study investigated the association of C49620T polymorphism of the ABCC8 gene with T2D and body lipids in a Nigerian population.

## Methods

### Study population and design

This study is part of a case-control study that recruited T2D patients and patients without diabetes (ND) who are 30 years old or above visiting Enugu State University Teaching Hospital (ESUTH), Enugu Nigeria. Patients considered as T2D patients were diagnosed according the IDF criteria [12] with at least 1 year history of diabetes. Non-diabetic control patients were those without T2D or hyperglycaemia. Participants included in the study were without any critical health condition or complications of diabetes and were not on hospital admission. Women who are breastfeeding or pregnant as well as HIV positive patients were excluded from the study. The sample size (*n*) was calculated according to Charan and Biswas [13] using the formula: $n=\frac{\ 2{\mathrm{SD}}^2\ {\left(\mathrm{Za}/2+{\mathrm{Z}}_{\mathrm{B}}\right)}^2}{{\mathrm{d}}^2}=74.19$.

Where; SD = standard deviation, Z_a/2_ = Zscore for type 1 error of 5%, Z_B_ = Zscore at 95% power and d = Effect size from previous study [14]. Thus, 74 participants were recruited for each arms (case and controls) making a total of 148 participants.

### Ethical considerations

The study was conducted in accordance with the Helsinki Declaration. Before the commencement of study, ethical clearance was obtained from ESUTH ethics committee (Approval No: ESUTHP/C-MAC/RA/034/174). Written informed consent was obtained from all patients before participation in the study.

### Data collection and biochemical analyses

Following recruitment, participants were administered a research questionnaire which sought relevant demographic information including age, sex, ethnicity, location and disease history. Systolic blood pressure (SBP) and diastolic blood pressure (DBP) were measured using an automatic sphygmomanometer. In addition, anthropometric parameters including body weight, height and waist circumference (WC) of participants were measured. Weight and height of participants were used for the estimation of Body Mass index (BMI) as weight (Kg)/ height^2^ (m^2^).

Fasting blood glucose (FBG) was measured from whole blood using an Accucheck glucometer [15]. Total cholesterol (TC), low density lipoprotein (LDL), triglyceride (TG) and high-density lipoprotein (HDL) were measured in serum using commercially available kits (Randox Laboratories Ltd., United Kingdom) following the manufacturer’s recommended protocol. TC was determined according to the enzymatic method of Allain and collaborators [16], TG was determined by the enzymatic method of Esders and Michira [17] and HDL by the precipitation method of Grove [18]. LDL was estimated using the Freidwald’s formula: LDL = TC- (TG/5) – HDL [19].

### Molecular genotyping of ABCC8 C49620T variant

DNA was extracted using the GeneJET Genomic DNA Purification kit (K0721) by Thermo Fisher Scientific Inc., USA following the manufacturer’s recommended procedure. The ABCC8 C49620T (rs1799854) gene was amplified in a PCR reaction as previously reported by He et al. [20], using primers with the following sequences; Forward - 5’-TTGGGTGCATCTGTCTGTCTGTCTTT-3′ and Reverse: 5’-AGCCCACCTGCCCCACGAT-3′. The PCR mix contained 8 μl of genomic DNA (< 10 ng), 12 μl of taq quick-load 2× master mix (New England Biolab (NEB), USA) and 4 μl of 50 μM of each primer. The PCR condition includes an initial denaturation at 94 °C for 5 min followed by 35 cycles of denaturation at 94 °C for 20s, annealing at 62 °C for 40s and extension at 72 °C for 40s with the final extension at 72 °C for 8 min. The 122 bp amplicon of *ABCC8* was visualised on 2% agarose gel (100 V for 20 min). Restriction digestion was achieved using 10 U *PstI* restriction enzyme (1ul, New England Biolabs, USA) for 6 h at 37 °C. Restriction fragments were resolved on a 3% agarose gel stained with ethidium bromide and visualized under UV light. The C49620 allele was cleaved into 88 and 34 bp restriction fragments, but T49620 allele remains uncleaved (122 bp).

### Statistical analysis

Data was analyzed using Statistical Package for Social Sciences (SPSS) version 16. Anthropometric, FBG and lipid profile data were expressed as frequencies or mean ± Standard Error of the Mean (S.E.M). Parametric independent sample *t*-test was used to compare mean differences of the lipid profile data and baseline study parameters for the various ABCC8 C/T genotypes of participants. Pearson chi-square (χ2) test was used to test for the Hardy-Weinberg equilibrium by comparing genotype and allele frequencies in the diabetic and non-diabetic subjects. Binary and multinomial logistic regression were used to determine the odd ratio (OR) by comparing allele and genotype frequencies between diabetic and non-diabetic patients. A confidence interval of 95% was taken and a *p*-value less than 0.05 was considered statistically significant.

## Results

**Fig. 1:**
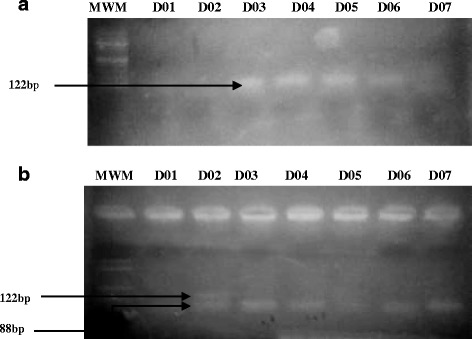
RFLP-PCR of ABCC8 C49620T Polymorphism. **a** shows the presence of the amplified gene for D01-D07 at 122 bp. **b** shows the restriction digest products of the gene. A 122 bp indicates the T allele while a 88 bp indicates the C allele. Sample D01 and D03-D07 show the presence of the wild-type homozygote CC genotype while D02 shows the presence of the heterozygote CT genotype. MWM indicates 100 bp molecular weight marker

In total, 73 T2D patients and 75 ND patients were recruited for the study. The demographic and clinical characteristics of the study population are summarized in our previous publication [21]. As summarized in Table 1, all 148 samples were successfully amplified for ABCC8 C49620T gene fragment with molecular size of 122 bp (Fig. 1a). Following restriction digestion, the expected product sizes were DNA fragment of 88 and 34 bp for normal homozygote CC genotype, fragments with 122 and 88 bp for a heterozygote CT genotype and an uncleaved band with size 122 bp for the mutant homozygote TT genotype (See Fig. 1b). The 34 bp was not seen on the gel. The mutant T allele was significantly (*p* < 0.05) predominant in both the ND and T2D patients than the wild C allele. Moreover, the prevalence of the T allele was greater in the T2D patients (33.1%) compared with their ND counterparts (28.0%). The homozygote CC and heterozygote CT genotypes were more prevalent in ND patients compared with T2D patients while the homozygote mutant TT genotype showed greater prevalence in T2D patients compared to ND patients. The CT genotype was not associated with T2D risk (*p*>0.05) while the TT genotype was associated with risk of T2D (OR: 2.58, 95% CI: 1.15-5.77, *p*<0.0021) when compared with the CC genotype after adjusting age. The distribution of the genotype frequencies between T2D patients and ND patients did not violate the Hardy-Weinberg equilibrium (χ2 = 2.78; *p* = 0.249).

**Table 1:** Association of ABCC8 C/T alleles and genotypes in participants

	ABCC8 C/T variant	T2D (%) patients	ND (%) patients	OR (95% CI)	*p*-value
Allele	C	48 (16.2)	67 (22.6)	–	
	T	98 (33.1)	83 (28.0)	1.7 (1.06-2.73)	0.028
	Total (2 N)	146 (49.3)	150 (50.7)		
Genotype	CC	19 (12.8)	27 (18.2)	–	
	CT	10 (6.8)	13 (8.8)	1.09 (0.39-3.01) 1.26 (0.44-3.60)^a^	0.863 0.668^a^
	TT	44 (29.7)	35 (23.6)	1.79 (0.86-3.73) 2.58 (1.15-5.77)^a^	0.122 0.021^a^
	Total (N)	73 (49.3)	75 (50.7)		

The results showed significant differences (*p*< 0.05) in allelic and genotypic frequencies between the T2D and ND patients after adjusting for age

*OR* odd ratio OR, *CI* confidence interval, *N* number of participants

^a^Indicates age adjustments of study participants

To determine the model that predicts the greatest risk (OR) of T2D, the codominant, dominant and recessive models were evaluated as shown in Table 2. The codominant model (CC vs. CT vs. TT) showed no significant risk of T2D (OR: 0.56 (0.27-1.17, *p* = 0.311) with the CT and TT genotypes compared along with CC genotype. The dominant model (CC vs. CT + TT = XT) with the T allele carriers (XT genotype) did not shown any significant risk of T2D when compared with the CC genotype (OR: 1.60, 95% CI: 0.79-3.23, *p* = 0.191). Similarly, the recessive model (CX = CC + CT vs. TT) did not show any significant risk of T2D when the TT genotype was compared to the CX genotype (OR: 1.73, 95% CI: 0.90-3.33, *p* = 0.098). However, following age adjustment, the mutant genotypes (CT and TT) showed significant risk (*p*<0.05) of T2D for all the models; the codominant, dominant and recessive models with the recessive model presenting the greatest risk (OR: 2.39, 95% CI: 1.16-4.91, *p* = 0.018) as shown in Table 2.

**Table 2:** Association of ABCC8 C/T genotypes in participants based on risk models

	ABCC8 C/T genotypes	T2D (%) patients	ND (%) patients	OR (95% CI)	*p*-value
Codominant model	CC	19 (12.8)	27 (18.2)	–	
	CT	10 (6.8)	13 (8.8)	–	
(CC vs. CT vs. TT)	TT	44 (29.7)	35 (23.6)	0.56 (0.27-1.17) 1.04 (1.01-1.07)^a^	0.311 0.009^a^
Dominant model (TX)	CC	19 (12.8)	27 (18.2)	–	
(CC vs. CT + TT = XT)	TT + CT	54 (36.5)	48 (32.4)	1.60 (0.79-3.23) 2.13 (1.00-4.52)^a^	0.191 0.05^a^
Recessive model (CX)	CC + CT	29 (19.6)	40 (27.0)	–	
(CX = CC + CT vs. TT)	TT	44 (29.7)	35 (23.6)	1.73 (0.90-3.33) 2.39 (1.16-4.91)^a^	0.098 0.018^a^
	Total (N)	73 (49.3)	75 (50.7)		

The results showed significant differences (*p*< 0.05) in genotype frequencies between the T2D and ND patients after adjusting for age for all models

*OR* odd ratio OR, *CI* confidence interval, *vs.* versus, *N* number of participants

^a^Indicates age adjustments of study participants

The relationship of ABCC8 (C/T) polymorphism with demographic and clinical characteristics of participants is summarized in Table 3. Comparison of ABCC8 C/T genotypes (CC, CT, TT) did not show any significant differences (*p*>0.05) on the anthropometric parameters (BMI and WC) of patients. The levels of FBG and lipid profile parameters (TC, TG and LDL) were not significantly different (*p*>0.05) among the various genotypes (CC, CT and TT) in the study population. However, HDL was significantly higher in patients with the mutant homozygote TT genotype (*p* = 0.005). To confirm the above finding, the relationship between ABCC8 (C/T) polymorphism with demographic and clinical characteristics was further assessed separately among T2D patients as well as ND patients as summarized in Table 4. Among non-diabetic patients, TC, TG and LDL levels were significantly lower (*p* < 0.05) while HDL was significantly higher (*p* < 0.005) in patients with the mutant TT genotype compared to the CT and TT genotypes. On the other hand, there were no significant differences (*p* > 0.05) of demographic and clinical characteristics for the various genotypes (CC, CT and TT) among type 2 diabetes patients.

**Table 3:** Relationship of ABCC8 (C/T) polymorphism with demographic and clinical characteristics of participants

	CC genotype	CT genotype	TT genotype	*p-value*
Age (year)	55.98 ± 2.16	53.57 ± 2.56	50.84 ± 1.62	0.148
WC (cm)	94.19 ± 2.66	97.00 ± 4.02	94.75 ± 1.99	0.836
BMI (Kg/m^2^)	29.24 ± 1.51	28.73 ± 1.25	30.05 ± 1.15	0.818
FBG (mg/dl)	119.69 ± 15.59	97.43 ± 12.28	118.92 ± 9.36	0.545
TC (mg/dl)	185.46 ± 20.52	217.42 ± 19.75	247.78 ± 25.75	0.206
TG (mg/dl)	192.12 ± 11.49	220.36 ± 18.23	187.64 ± 15.09	0.470
LDL (mg/dl)	118.61 ± 20.64	132.02 ± 20.71	161.90 ± 26.39	0.460
HDL (mg/dl)	36.11 ± 2.47	41.88 ± 3.98	57.38 ± 5.23	0.005

Results showed a significantly higher (*p* < 0.05) HDL-c level in patients with TT genotype. Results are expressed as mean ± S.E.M

*S.E.M* standard error of the mean, *WC* waist circumference, *BMI* body mass index, *FBG* fasting blood glucose, *TC* total cholesterol, *TG* triglyceride, *LDL* low density lipoprotein, *HDL* high density lipoprotein

**Table 4:** Comparison of ABCC8 (C/T) polymorphism with demographic and clinical characteristics among T2D and ND patients

	Type 2 Diabetes patients				Non-Diabetic patients			
	CC genotype	CT genotype	TT genotype	*p-value*	CC genotype	CT genotype	TT genotype	*p-value*
Age (year)	58.67 ± 2.37	61.50 ± 2.59	55.05 ± 1.55	0.128	54.19 ± 3.22	47.46 ± 3.19	45.53 ± 2.89	0.111
WC (cm)	103.12 ± 2.89	104.00 ± 2.50	98.32 ± 2.37	0.342	88.12 ± 3.56	90.70 ± 6.86	89.94 ± 3.26	0.608
BMI (Kg/m^2^)	32.74 ± 3.09	29.31 ± 1.86	31.29 ± 1.83	0.764	26.82 ± 1.25	28.25 ± 1.76	28.46 ± 1.16	0.909
FBG (mg/dl)	203.99 ± 27.91	150.30 ± 13.73	153.19 ± 13.35	0.128	60.37 ± 3.18	56.77 ± 7.96	73.94 ± 7.26	0.146
TC (mg/dl)	259.88 ± 43.84	235.58 ± 10.16	318.20 ± 43.86	0.516	135.84 ± 10.65	203.46 ± 34.17	161.27 ± 5.29	0.010
TG (mg/dl)	224.97 ± 15.33	286.25 ± 19.69	237.82 ± 23.97	0.482	170.22 ± 14.96	169.68 ± 19.01	124.55 ± 7.51	0.010
LDL (mg/dl)	183.44 ± 46.36	141.22 ± 11.43	241.07 ± 44.22	0.445	75.39 ± 9.25	124.93 ± 36.14	64.64 ± 6.64	0.010
HDL (mg/dl)	31.12 ± 4.36	38.31 ± 7.02	33.46 ± 2.62	0.612	39.43 ± 2.81	44.63 ± 4.66	87.45 ± 9.11	<0.0001

Results showed a significantly (*p* < 0.05) lower TC, TG, LDLand higher HDL levels in non-diabetic patients with TT genotype. Results are expressed as mean ± S.E.M

*S.E.M* standard error of the mean, *WC* waist circumference, *BMI* body mass index, *FBG* Fasting blood glucose, *TC* total cholesterol, *TG* triglyceride, *LDL* low density lipoprotein cholesterol, *HDL* high density lipoprotein cholesterol

## Discussion

Insulin resistance, insulin secretion and obesity are the most prevalent underlying causes of T2D and thus, much interest has been to identify potential related genes involved and possible mutations that may confer susceptibility to the disease. Polymorphisms of the ABCC8 gene which encodes SUR1, a surface receptor for sulfonylurea that regulates insulin secretion, has been investigated with much concern on the C49620T variant which has shown to be associated with T2D [22]. Our study showed predominance of the T allele over the C allele with a significant risk (*p* < 0.05) of T2D. This is in contrast to findings from other regions across the globe where the wild type C allele is usually predominant in the population [23, 24]. The homozygote mutant TT genotype was more frequent than the homozygote wild type CC genotype while the heterozygote CT was the least frequent. The TT genotype was more frequent in T2D patients compared with their ND counterparts and vice versa for the CC genotype. When the mutant genotypes (TT, TT + CT) were compared with the CC genotype between T2D patients and ND patients, there was no significant risk (*p* < 0.05) of T2D for the dominant, codominant and recessive models. However, when these models were adjusted for age, the risk of the mutant genotype to cause T2D was significant (*p*< 0.05). This result suggests that the mutant genotypes TT and CT have possible risk of predisposing people to T2D. This finding is consistent with previous reports on the association between ABCC8 C/T polymorphism and T2D [25, 26] as well as the association between this polymorphism and gestational diabetes [27] together with the attendant effects on insulin secretion [7, 8]. However, this study is in contrast to a large-scale association studies which showed no association of the polymorphism with T2D [28]. The recessive model predicted the greatest risk for T2D and suggests that the mutant TT genotype is the most vulnerable genotype with a greaterrisk of T2D than the CT and CC genotypes. The distribution of genotypes between T2D patients and ND patients did not violate the Hardy-Weinberg equilibrium in this study (χ2 = 2.781; *p* = 0.249), thus the population under consideration was stable.

Since insulin has diverse functions in the body regulating carbohydrates and lipids by stimulating lipid synthesis from glucose and prevents lipid degradation or lipolysis [29], we evaluated the relationship between the ABCC8 C/T polymorphism with obesity, cholesterol, triglyceride and lipoproteins. Findings from our study showed no significant differences (*p*> 0.05) of these genotypes (CC, CT and TT) with anthropometric or obesity parameters (BMI, WC), FBG and serum lipids (TC, TG and LDL) in the study population. This confirms findings of previous studies which also showed no association of this polymorphism with anthropometric parameters [23], obesity and body fat [22]. However, HDL level was significantly higher (*p* < 0.05) in patients with the TT genotype compared to those with CT and CC genotypes in the study population. Further comparison of the clinical and demographic characteristics on the various genotypes (CC, CT and TT) among non-diabetic patients, showed TC, TG, LDL to be significantly lower (*p* < 0.05) while HDL was significantly higher (*p* < 0.05) in patients with the mutant TT genotype. If similar finding is observed for T2D patients, it may suggest that the TT genotype may improve the HDL level as well as reduce TC, TG and LDL levels in patients, thereby improving lipid abnormalities which may be beneficial to T2D as HDL is known to play essential role in the removal of free cholesterol from blood thereby reducing T2D susceptibility [30]. However, no significant differences (*p* > 0.05) were observed for the demographic and clinical characteristics among type 2 diabetes patients. Thus, it is not yet conclusive to propose that TT genotype may improve the diabetic status of patients by reducing lipid abnormalities as such finding was not observed among type 2 diabetes patients in this study. Further detailed studies are needed to better explain or confirm the association of ABCC8 C/T polymorphic genotypes with T2D as well as body lipids taking into consideration that the sample size of this study may be limiting to make generalized conclusions.

## Conclusion

This study revealed possible association of ABCC8 C49620T polymorphism with T2D marked by predominance of the mutant T allele and TT genotype in T2D patients. However, the relationship between TT genotype and lipid abnormalities for possible beneficial effect on people suffering from T2D is not clear. Thus, further studies are needed to further investigate this possibility.

## Abbreviations

ABCC8: ATP binding cassette, subfamily C, member 8
BMI: Body Mass index
bp: Base pair
CI: Confidence interval
ESUTH: Enugu State University Teaching Hospital
HDL: High density lipoprotein
HIV: Human immunodeficiency virus
KATP: ATP-sensitive potassium channel
LDL: Low density lipoprotein
ND: Non-diabetic
OR: Odd ratio
RFLP-PCR: Restriction fragment length - Polymerase Chain Reaction
S.E.M: Standard error of the mean
SPSS: Statistical Package for Social Science
SUR: Sulfonylurea receptor
T2D: Type 2 diabetes
TC: Total cholesterol
TG: Triglyceride
UD: Undigested sample
WC: Waist circumference
WHO: World Health Organisation
